# Health-Promoting Properties of Proanthocyanidins for Intestinal Dysfunction

**DOI:** 10.3390/nu12010130

**Published:** 2020-01-02

**Authors:** Carlos González-Quilen, Esther Rodríguez-Gallego, Raúl Beltrán-Debón, Montserrat Pinent, Anna Ardévol, M Teresa Blay, Ximena Terra

**Affiliations:** MoBioFood Research Group, Departament de Bioquímica i Biotecnologia, Universitat Rovira i Virgili, 43007 Tarragona, Spain; carlosalberto.gonzalez@urv.cat (C.G.-Q.); esther.rodriguez@urv.cat (E.R.-G.); raul.beltran@urv.cat (R.B.-D.); montserrat.pinent@urv.cat (M.P.); anna.ardevol@urv.cat (A.A.); ximena.terra@urv.cat (X.T.)

**Keywords:** gut, permeability, inflammation, metabolic endotoxemia, obesity, IBD, flavonoid, flavan-3-ol, condensed tannin, procyanidin

## Abstract

The intestinal barrier is constantly exposed to potentially harmful environmental factors, including food components and bacterial endotoxins. When intestinal barrier function and immune homeostasis are compromised (intestinal dysfunction), inflammatory conditions may develop and impact overall health. Evidence from experimental animal and cell culture studies suggests that exposure of intestinal mucosa to proanthocyanidin (PAC)-rich plant products, such as grape seeds, may contribute to maintaining the barrier function and to ameliorating the pathological inflammation present in diet-induced obesity and inflammatory bowel disease. In this review, we aim to update the current knowledge on the bioactivity of PACs in experimental models of intestinal dysfunction and in humans, and to provide insights into the underlying biochemical and molecular mechanisms.

## 1. Introduction

The primary function of the intestinal tract is to digest food components and absorb nutrients and water from the lumen to the systemic circulation. The intestine is also a physical barrier that is in contact with the environment. As a result, the intestinal epithelium is constantly exposed to potentially pathogenic microorganisms, toxins, and harmful components of the diet. When there are disturbances in the barrier function and mucosal immune homeostasis, the influx of intestine luminal content triggers an exaggerated mucosal immune response [1]. Ultimately, chronic exposition to these detrimental environmental stimuli may lead to the development of local and systemic inflammatory conditions [2,3].

Natural products have been recognized as a source of therapeutic agents for many years [4]. Some plant-derived phenolic compounds show promising anti-inflammatory effects and have been associated with the prevention of certain chronic diseases [5]. Proanthocyanidins (PACs), also known as condensed tannins, are oligo- and polymeric end products of the flavonoid biosynthesis pathway in plants [6]. There has been extensive laboratory research into the effects of both pure PAC molecules and PAC-rich extracts on overall health. These phytochemicals show a wide range of physiological activities [7], including anti-inflammatory and barrier protective effects in the intestine [8,9,10], which may be interesting in the context of diet-induced obesity and inflammatory bowel disease (IBD).

We have reported previously that grape-seed PACs and other flavonoids have beneficial effects on inflammation [11,12,13] and protect the intestine against alterations associated with diet-induced obesity in rats [8,9,14,15]. In addition, research conducted during the last decade with cell culture and animal models has made significant progress in determining the underlying mechanism of the health promoting properties of PACs in the gastrointestinal tract and peripheral tissues.

## 2. The Intestinal Barrier

The intestinal epithelium is a single cell-layer responsible for separating underlying mucosal tissues from the environment and is the largest exposed surface area in the body [16]. As there is a prolific commensal microbial community in the intestinal lumen (intestinal microbiota), epithelial integrity plays a pivotal role in maintaining overall health [16,17]. The intestinal epithelium is integrated by several cell types with specialized functions. The enterocytes are responsible for the absorptive function and constitute the most abundant epithelial cell lineage. The goblet cells are implicated in the synthesis of secretory mucin glycoproteins that form the mucus layer, with mucin 2 (MUC2) being the most prominent [18]. Other cellular types integrating the epithelium, microfold (M) [19], Paneth, and enteroendocrine cells are specialized in antigen sampling and presentation to dendritic cells, synthesis of antimicrobial peptides, and secretion of hormones, respectively.

The first strategy the host tissue has to maintain is its homeostatic relationship with the intestinal microbiota to minimize the physical interaction with microorganisms, thus limiting microbial translocation and physiological inflammation [20,21]. The thick mucus layer secreted by goblet cells represents a primary defense line against environmental insults [18]. In addition, the enterocytes are joined together forming an intricately and well-regulated barrier sustained by intercellular junctions linked to the cell cytoskeleton, such as tight junctions (TJs), desmosomes, and adherent junctions. TJs partially seal the paracellular space and prevent passive transport of large molecules, including microbial components and other potentially pro-inflammatory agents [1,22]. The main protein components of TJs are claudins, occludins, and junction adhesion molecules (JAMs), which are associated with peripheral proteins such as zonula occludens (ZOs) [22].

## 3. Intestinal Inflammatory Response: The Critical Role of TLRs and NF-κB

Beneath the intestinal epithelium, a thin mucosal layer of connective tissue known as the lamina propria hosts the gut-associated lymphoid tissue (GALT), which is responsible for the intestinal immunological response to microbial and non-microbial antigens [23]. GALT is distributed along the intestinal tract and includes aggregates of lymphoid cells forming scattered structures such as Peyer’s patches, which occur mainly in the ileum [23,24]. In the follicle-associated epithelium (FAE), M cells actively internalize, process, and present microbial and non-microbial antigens taken from the lumen [19]. M cells also contribute to the induction of antigen-specific immunoglobulin A (IgA), the dominant isotype in mucosal tissues [19].

The constant exposure of the intestine epithelium to the microbiota generates the need for a homeostatic balance between tolerance and the immune response [21]. Thus, intestinal immune cells exert protective immunity against pathogens while they show a limited responsiveness to commensal bacteria and food derived antigens. Failure to maintain this balance may lead to inflammatory conditions [25]. Inflammation is a normal biological response of the immune system triggered by detrimental stimuli or conditions, such as infections and tissue damage. The inflammatory response is the first of several overlapping processes that lead to tissue repair and regeneration [26]. During the inflammatory response, cell signaling pathways are activated to regulate the concentration of pro-inflammatory mediators in the affected tissue and the recruitment of immune cells from the circulation [27].

The innate immune response is initiated by pattern recognition receptors (PRRs) recognizing microbial antigens known as pathogen-associated molecular patterns (PAMPs). Among others, the PRRs include Toll-like receptors (TLRs) located on the cell membrane and nucleotide binding oligomerization domain (NOD)-like receptors (NLRs) present in the cytoplasm. PRRs are the primary component of innate immunity responsible for preventing systemic dissemination of pathogens by activating pro-inflammatory intracellular signaling pathways [28]. In addition, TLRs can recognize multiple endogenous molecules derived from damaged tissue (damage-associated molecular patterns or DAMPs) such as peptides, lipids, glycans, and nucleic acids [29]. PRR expression is not uniformly distributed along the proximal/distal axis of the intestinal tract [30,31] or on the crypt/villus axis of the epithelium [30]. In fact, there is a significantly higher expression of TLRs in the distal segments of the intestine (ileum and colon) where the microbial population is enriched [30,31]. Moreover, a higher PRR expression is found along the crypt/villus axis of the epithelium in both villus and lamina propria [30].

Activation of TLRs (mainly TLR4/myeloid differentiation 2 (MD-2) complex) by bacterial lipopolysaccharides (LPS), induces recruitment and phosphorylation of multiple intracellular protein complexes, leading to the stimulation of the nuclear factor kappa-light-chain-enhancer of activated B-cell (NF-κB) signaling [32]. NF-κB is a family of inducible transcription factors responsible for expression of numerous genes implicated in the innate immune response, including cytokines such as the tumor necrosis factor-alpha (TNF-α), chemokines, adhesion molecules (CAMs), and inducible enzymes such as the nitric oxide synthase (iNOS) and cyclooxygenase 2 (COX-2) [33]. Proteins p50 (NF-κB1), p52 (NF-κB2), RelA (p65), RelB, and c-Rel are present in the cytosol as inactive homo- or heterodimers bound by a family of inhibitory proteins known as inhibitors of κB (IκB), of which IκBα is the most studied member. NF-κB is activated by both canonical signaling, associated with the innate immune response, and non-canonical signaling, implicated in maturation, survival, and homeostasis of B cells [34]. In the canonical signaling pathway, TLR ligands activate the IκB kinase (IKK) complex. IKK induces site-specific phosphorylation of IκB that leads to its ubiquitination and proteasomal degradation, releasing NF-κB dimers, predominantly p50/RelA complex. Subsequent activation of this complex by post-translational modifications promotes its translocation to the nucleus where transcription of target genes is induced [33]. Activation and differentiation of naive T cells are processes of adaptive immunity also influenced by gene programs regulated by NF-κB [35,36]. Depending on the cytokines present during activation, CD4^+^ T cells differentiate in multiple subsets of T helper cells with different cytokine profiles. Th1 cells play a role in the immune response against intracellular pathogens, essentially through the production of interferon (IFN)-γ. Th2 cells are involved in the response to extracellular pathogens and allergic reactions and are producers of interleukin (IL)-4, IL-5, IL-6, IL-9, IL-13, and IL-25 [35]. Th17 cells primarily express IL-17 and are also related to the immune response against extracellular pathogens [36].

TLRs are also required for production of IgA, which in turn contributes to determining the microbiota composition and maintenance of the non-inflammatory host-microbial relationship [37]. Furthermore, TLR2 signaling is associated with the regulation of TLR3/4-mediated production of pro-inflammatory Th1 cytokines through anti-inflammatory IL-10 induction [38]. TLR2 activation also improves the barrier function by means of protein kinase C-mediated sorting and assembly of ZO-1 [39].

## 4. Cytokine-Induced Regulation of Tight Junctions

TJ protein complexes are not rigid but rather exhibit dynamic elements that ultimately modulate the epithelial paracellular permeability [40]. Phosphorylation of the myosin II regulatory light chain in the perijunctional actomyosin ring by myosin light chain kinase (MLCK), produces a transient TJ opening that enhances paracellular flux [40]. MLCK activation is also implicated in the redistribution of ZO-1 [41] and caveolin-1-dependent occludin endocytosis [42]. TJ protein expression and MLCK activation are both regulated by multiple signaling molecules, including cytokines produced as a result of the activation of NF-κB. These regulatory processes are complex and also involve the activation of multiple kinase enzymes downstream, such as mitogen-activated protein kinases (MAPKs), phosphoinositide-3-kinases (PI3Ks), and monophosphate-activated protein kinase (AMPK) [43]. For example, IFN-γ down-regulates expression of ZO-1 and occludin in an AMPK-dependent pathway [44]. The activation of MLCK by interleukin IL-1β is mediated by p38 MAPK-dependent induction of the transcription factor ATF2, which binds to the MLCK promoter [45]. The activation of MLCK induced by TNF-α is mediated by extracellular signal-regulated kinases (ERK1/2), which phosphorylates and promotes nuclear translocation of the transcription factor ELK-1 [46]. TNF-α also induces expression of the pore-forming claudin-2 through the upregulation of the transcription factor CDX2 mediated by PI3K/protein kinase B (Akt) signaling [47].

## 5. Intestinal Dysfunction: Pathophysiological Basis and Environmental Triggers

**Figure 1:**
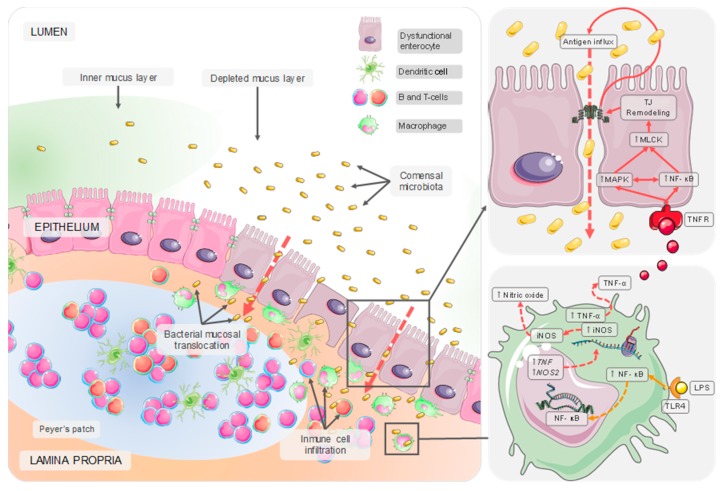
Dysfunctional intestinal mucosal. Chronic exposition to detrimental environmental stimuli, including several food components, may lead to dysbiosis, mucus layer depletion, and breakdown of the epithelial barrier. Constitutive stimulation of NF-κB signaling by bacterial endotoxins induce overproduction of pro-inflammatory cytokines and reactive species of oxygen and nitrogen, feeding back the epithelial barrier disruption and immune cell infiltration.

We define intestinal dysfunction as the state of increased intestinal permeability and pathological inflammation seen in diet-induced obesity and IBD (ulcerative colitis and Crohn’s disease) [48]. Evidence suggests that changes in the epithelial barrier function and inflammation are associated with metabolic disorders and alterations in the regulation of body weight [49,50,51]. This pathological inflammation is characterized by increased levels of pro-inflammatory mediators and enhanced infiltration of immune cells into the intestinal tissues ([52,53] and Figure 1). Regardless of the specific triggering events, intestinal inflammatory states share common immunological processes. However, intestinal dysfunction associated with IBD is significantly more severe than in diet-induced obesity [48]. Diet-induced obesity and Crohn’s disease lead to a Th1 response with increased IFN-γ production and reduced Th17 response [53,54], while ulcerate colitis has often been considered a Th2-mediated disease [53].

Multiple environmental factors have been identified as potential triggers of intestinal inflammatory conditions, including Western dietary habits [55]. It has been described that saturated fats play a direct role in inflammatory signaling. Saturated fatty acids (SFA) such as lauric (C12:0) and palmitic (C16:0) directly induce NF-κB activation, acting as non-microbial TLR2 and TLR4 agonists in macrophages [56]. Data suggest that activation of TLRs by SFA is mediated by TLR dimerization and recruitment into lipid rafts [57]. We have reported mild intestinal inflammation and increased permeability in rats feeding on a cafeteria diet consisting of high-saturated fat/high-refined sugar food products [58]. This enhanced permeability has been shown to favor bacterial LPS and other potentially pro-inflammatory molecules entering the systemic circulation, which is known as metabolic endotoxemia [15].

Diet plays an important role in the composition of intestine microbiota, promoting or inhibiting growth of microorganisms [59]. Alterations in the composition and metabolism of the intestinal microbiota (dysbiosis) have also been associated with the consumption of high-saturated fat diets in rodents and humans [60,61]. In fact, metagenomic analysis of the intestinal microbiome in Western populations has shown a reduction not only of microbial diversity but also of functional potential [62]. Dysbiosis is linked to obesity-associated intestinal inflammation, although the “egg or hen” question related to the cause-effect relationship is not answered yet [63]. High-fat intake in rodents often decreases overall diversity of microbiota and the abundance of *Bacteroidetes*, and increases the relative abundance of *Firmicutes* [64,65]. Several human studies have described similar associations [66,67], but the importance of the ratio *Firmicutes* to *Bacteroidetes* remains controversial [68,69], and some authors state that the experimental results are not sufficiently consistent [70]. Interestingly, the existence of a colitogenic microbiota was demonstrated in T-bet^−/−^ × RAG2^−/−^ deficient mice whose spontaneous ulcerative colitis was horizontally transmissible to wild-type individuals when co-housed [71]. Although mechanisms by which dysbiosis trigger intestinal inflammation are not fully understood, it is known that they involve the loss of immune tolerance due to local immune homeostasis disruption and continuous abnormal activation of TLRs [72]. Overactivation of NF-κB leads to a chronic pro-inflammatory cytokine release and immune cell infiltration [33]. Furthermore, as stated before, pro-inflammatory cytokines such as TNF-α and IFN-γ decrease barrier integrity, feeding back the dysfunctional state. The association of NF-κB signaling with intestinal inflammation-related pathologies is supported by the NF-κB-inhibiting properties of drugs used in the treatment of IBD, such as aminosalicylates [73,74]. Given the importance of NF-κB in inflammation-associated pathologies, it is relevant to study dietary components that negatively regulate the NF-κB signaling pathway, such as PACs and other flavonoids [9].

## 6. PACs: Chemical Structure, Occurrence, and Intake

**Figure 2:**
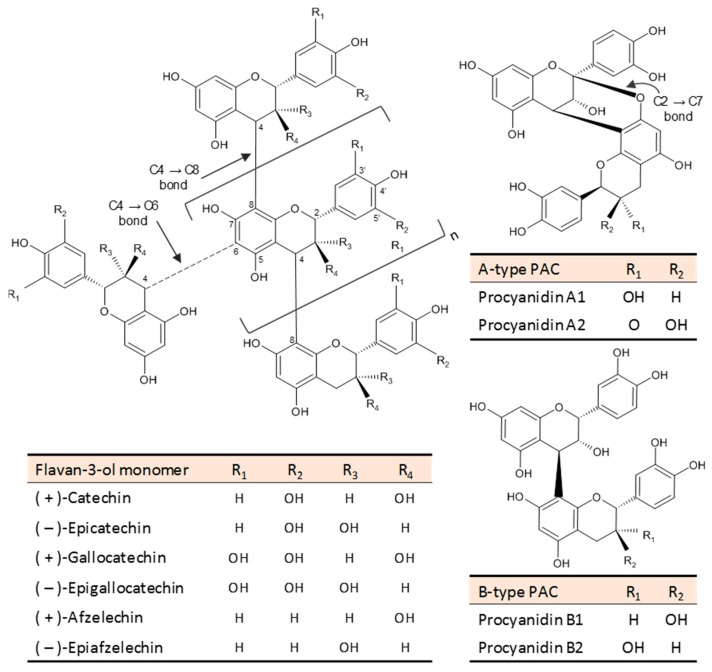
Chemical structures of proanthocyanidins (PACs). Flavan-3-ol monomers differ based on the hydroxylation pattern and their cis- or trans- configuration. Dimers A1/A2 and B1/B2 are shown as example of A- and B-type PACs, respectively.

PACs consist of flavan-3-ol subunits with a degree of polymerization (DP) equal to or greater than 2, mainly linked by (4 → 8) or (4 → 6) carbon-carbon bonds (B-type PACs) [75]. In some botanical sources an additional (2 → 7) ether-linkage also occurs (A-type PACs) [76] (Figure 2). Depending on the type of monomers, PACs can be classified into procyanidins, prodelphinidins, and propelargonidins. The most abundant group, procyanidins, consists exclusively of (+)-catechin and (−)-epicatechin monomers [77]. Prodelphinidins and propelargonidins are composed of (−)-gallocatechin/(−)-epigallocatechin and (+)-afzelechin/(−)-epiafzelechin monomers, respectively [75], and have a more limited distribution.

Dietary assessment studies have shown that PACs, especially procyanidins, are among the most abundant polyphenols in the human diet [6], as they are present in a variety of botanical sources and plant food products such as tea, fruits, nuts, cacao products, legumes, and cereal grains [1,2]. However, PAC intake varies widely between geographical regions and cultures and is greatly dependent on eating habits, lifestyle behaviors, and socioeconomic status [78]. The daily PAC (dimers to polymers) intakes in adult populations from Korea, the U.S., Mexico, and EU were estimated as 71 [79], 73 [78], 103 [80], and 123–180 mg [81,82], respectively, but intakes up to 230 mg d^−1^ have been reported in some regions of Spain and Norway [83].

## 7. The Fate of PACs after Ingestion

Flavan-3-ols are remarkably stable during gastric transit in humans [84]. Monomers such as (+)-catechin and (−)-epicatechin are readily absorbed in the upper sections of the small intestine [85,86], recognized as xenobiotics and then subjected to an extensive phase II metabolism that generates glucuronidated, sulfated, and methylated conjugates [87]. Flavan-3-ol monomers and their conjugated metabolites reach peak plasma concentration 1–4 h after flavan-3-ol-rich food consumption [88,89,90]. Studies conducted in cultivated epithelial monolayers [91,92,93], rats [94,95], and humans [90,96] indicate that PAC absorption is conversely more limited and is highly dependent on DP, and that the permeation of larger oligomers (DPB > 5) and polymers is negligible. No PAC transporter has been identified in the enterocyte membrane in the small intestine. Thus, dimers to tetramers are passively transported across the intestinal epithelium essentially by paracellular diffusion. Although transcellular passive diffusion is not likely to occur due to the hydrophilic nature of PACs conferred by the multiple hydroxyl groups, uptake might be possible by endocytic mechanisms [92].

In humans, a study assessed the contribution of the ingested cocoa flavan-3-ols and procyanidins to the systemic pool, and found that the plasma (−)-epicatechin came from the orally administered cocoa (−)-epicatechin and not from their oligomers or polymers [97]. This is in agreement with evidence obtained with rats that suggests that PACs from different sources do not depolymerize to monomers after ingestion [98,99]. Stalmach et al. [86] conducted a study with ileostomized patients who were administered green tea, and found 70% of the ingested flavan-3-ol in the ileal fluid after 24 h. Altogether, these findings suggest that substantial amounts of ingested flavan-3-ol monomers and PACs remain unabsorbed in the small intestine and reach the colon. There, they are efficiently transformed by the colonic microbiota into low molecular weight phenolic compounds that can be absorbed by colonocytes [87].

In vitro fermentation of purified procyanidin dimers with human fecal microbiota has shown to produce mainly 2-(3′,4′-dihydroxyphenyl) acetic acid and 5-(3′,4′-dihydroxyphenyl)-γ-valerolactone [100]. In agreement with this, a randomized cross-over study in healthy humans found that a great portion of the ingested (−)-epicatechin and procyanidin B1 was metabolized by the colonic microbiota to produce phenyl-γ-valerolactones as the major microbial metabolites [90]. In this study, microbial degradation of larger procyanidins was substantially lower, possibly to the inhibition of digesting enzymes or to the antibacterial properties exhibited by these compounds. Other human studies analyzing the bioavailability of flavan-3-ols reported high levels of phenyl-γ-valerolactones in the circulation and urinary excretion after ingestion of a red grape pomace drink [101] and apple juice [102]. In the colonocytes and hepatocytes, these microbial products undergo further metabolism by phase II enzymes to produce conjugated derivatives. Margalef et al. [103] analyzed the tissue distribution of metabolites derived from a grape-seed proanthocyanidin extract (GSPE) 2 h after ingestion by rats. These authors detected a few microbial metabolites (methyl conjugated phenols) at low concentrations in the colon tissue, while most phase II metabolites (glucuronidated and methyl-glucuronidated forms) were found in the kidneys and liver. In humans, the major contributors to the excretion of phenyl-γ-valerolactones after ingestion of a red grape pomace drink, are sulphated and glucuronidated conjugates of 5-(3′,4′-dihydroxyphenyl)-γ-valerolactone [101].

## 8. Studies on the Benefits of PACs for Intestinal Dysfunction

During the last decade, the beneficial properties of PACs for intestinal function have been reported in several studies performed with cell culture models and experimental animals (Table 1 and Table 2). This experimental data indicate that PACs contribute to maintaining the intestinal barrier and improving mucosal inflammation induced by environmental insults. However, there are few studies on the effect of PACs on human intestinal health, although epidemiological studies connect PAC-rich food consumption with a lower risk of colorectal cancer [104].

In vitro models of inflammation have been fundamental in the comprehension of cellular mechanisms driving physiological effects of bioactive molecules. Studies on intestinal dysfunction have employed human colon carcinoma cell lines, with Caco-2 being the most well-established and widely used model of the human intestine barrier ([105] and Table 1). Mucus producer [106], macrophages [107], and B cell lines [108] have been employed in co-culture systems to explore the interaction between cell populations. Although there is a strong trend in the industry towards replacing animal experiments with human cell-culture based models [109,110], there are no in vitro models of the human intestine that replicate the complex interplay between cell types and the regulation of the barrier function by the mucosal innate and adaptive immunity. Therefore, most physiologically relevant data on intestinal dysfunction comes from the animal model. Most in vivo studies testing the effect of PAC supplementation on intestinal health have been performed in diet-induced obesity models and chemical-induced colitis models. The first resemble intestinal alterations seen in humans with metabolic syndrome [58]. The latter closely mimic histopathological features of human colitis and are frequently used to study the pathophysiology of IBD and the effectiveness of novel therapeutic drugs [111]. Notably, PAC-rich grape-seed extracts (GSPE) are between the most studied botanical extracts, mainly by in vivo approaches in rodents (Table 2).

### 8.1. Studies with Cell Culture Models

The data available on the interaction between PACs and permeability and inflammation markers in cell models of intestinal dysfunction are summarized in Table 1. Caco-2-based models have shown to be responsive to pro-inflammatory stimulation, producing a wide range of inflammatory mediators and increasing the paracellular permeability. Pro-inflammatory agents such as LPS, phorbol 12-myristate 13-acetate (PMA), and cytokines (TNF-α and IL-1β) have been used in multiple studies testing the effect of PAC molecules and PAC-rich botanical extracts on inflamed Caco-2 cells [10,112,115,116]. Stimulated-Caco-2 cell monolayers incubated with PACs generally show a reduction in gene expression and secretion of TNFα, IL-6, and IL-8 [10,112,113,115], which is often linked to the downregulation of NF-κB signaling at different levels [10,114,115]. An increased expression of antioxidant enzymes, such as glutathione peroxidase (GPx), superoxidase dismutase (SOD), and hemeoxygenase 1 (HO-1), has also been reported [10].

When permeable support systems such as transwell or Ussing chamber (UCh) are used, alterations in barrier integrity and paracellular permeability of epithelial cell monolayers are evaluated by transepithelial electrical resistance (TEER), an electrophysiological parameter that measures ion conductance across the monolayer, and by the transepithelial transport of molecular markers such as Lucifer yellow (LY) and fluorescently-labelled dextrans (FD) [125,126]. Some in vitro studies have associated PACs with increased TEER and decreased transport of permeability markers in the context of barrier dysfunction [115,116,117]. The expression levels of TJ proteins (claudins, occludins, and ZOs) often correlate, but not always [106], with intestinal permeability and are also considered markers of epithelial integrity. Bitzer et al. [116] found that the dextran sodium sulphate (DSS)-induced loss of barrier function in Caco-2 cells was significantly inhibited by polymeric PACs of cocoa but not by oligomers. Moreover, a higher barrier protective activity was determined in PACs with DP ≥ 7, which were able to reduce the detrimental effect of DSS in a dose-dependent fashion [116]. Effectiveness of procyanidin B2 ameliorating dextran sodium sulphate (DSS)-induced permeability alterations was examined using a Caco-2/HT29-MTX co-culture model [106]. Although procyanidin B2-incubated cells showed increased levels of the TJ proteins claudin-7, occludin, and ZO-1, these changes did not reduce TEER loss. Altogether, these results suggest that the ability of PACs to strengthen the intestinal barrier integrity depends on the degree of polymerization (DP).

### 8.2. In Vivo Studies of Diet-Induced Intestinal Dysfunction

The cafeteria (CAF) diet is a self-selected high-saturated fat/high-refined sugar diet that stimulates hyperphagia and a rapid weight gain in experimental animals [127,128]. In this feeding regime, highly palatable and energy dense foods commercially available, such as muffins, biscuits, bacon, sausages and sugared milk, are offered ad libitum [15,129]. A long-term CAF diet (62% carbohydrate (mostly simple sugar), 23% lipid, and 13% protein in dry matter) has negative effects on intestinal function in rodents, increasing intestinal permeability and inducing mucosal inflammation [58]. We have described the beneficial effects of administering GSPE against the intestinal dysfunction induced by a long-term CAF diet (17–18 weeks) in Wistar rats [8,14,15]. The composition of the GSPE used in these studies has been analyzed in detail and is shown in Table 3. Both nutritional (5–50 mg kg^−1^ [14]) and pharmacological (100–500 mg kg^−1^ [8,15]) doses of GSPE administered orally as a preventive [8] or counteractive treatment [14,15] tended to reduce intestinal inflammatory markers such as TNF-α release or myeloperoxidase (MPO) activity (an indicator of neutrophil infiltration in tissues). The reduction of plasma ovalbumin (OVA), an in vivo marker of intestinal permeability, was supported by (1) the increase in TEER in small and large intestine segments. This parameter is determined ex vivo by UCh-based protocols [8,15]. And (2) by the upregulation of TJ proteins such as ZO-1 [14] and claudin-1 [8,15]. Notably, the protective effect of GSPE in the intestinal barrier function was linked to the amelioration of metabolic endotoxemia (reduction of plasma LPS) and systemic inflammation (reduction of plasma TNF-α) in obese rats [15,130]. Other authors have also reported the upregulation of ZO-1 and claudin-1 TJ proteins in high-fat fed rats supplemented with other PAC-rich extracts [118].

### 8.3. In Vivo Studies of Chemical-Induced Intestinal Dysfunction

Chemical agents administered orally to induce colitis in rodents include trinitrobenzene sulphonic acid (TNBS) and DSS. These agents erode the colonic mucosal lining and produce the loss of the intestinal barrier function and colonic inflammation. In these models, the severity of outcomes depends on the dose of the chemical agent and the frequency of administration. Li et al. [132] found that intragastric administration of GSPE in rats at pharmacological doses (100–400 mg kg^−1^ d^−1^) prior to TNBS-induced recurrent colitis reduced weight loss and attenuated macro and microscopic tissue damage scores in the colon. This protective effect was accompanied by a reduction in oxidative stress (malondialdehyde; MDA), inflammation (IL-1β), and neutrophil infiltration (MPO activity) in colonic tissues. Remarkably, the beneficial effects of low-to-high doses of GSPE were comparable to those of sulfasalazine (200 mg kg^−1^ d^−1^), a potent inhibitor of NF-κB. Subsequent studies carried out by these authors with the same model confirmed the role of the GSPE down-regulating NF-κB response [120,121]. Amelioration of the redox status due to the increase in GPx and SOD activity was also observed in the colon tissues of GSPE-treated rats [120]. A preventive effect of procyanidin B2 was also evidenced in a mouse model of DSS-induced colitis [122]. Administration of procyanidin B2 (10–40 mg kg^−1^ d^−1^) attenuated the severity of tissue damage in the colon and reduced the levels of matrix metalloproteinase-9 (MMP-9), a marker of macrophage infiltration. In addition, inhibition of the NF-κB signaling and of NLRP3 inflammasome activation was observed, with a concomitant reduction in the gene expression of proinflammatory cytokines. Overall, the benefits of procyanidin B2 administration, especially at the highest dosage (40 mg kg^−1^), were comparable to those of mesalazine (200 mg kg^−1^), a cyclooxygenase (COX) inhibitor. The authors suggest that these effects were largely due to the reduction in activated macrophages infiltrating colonic tissues, probably driven by reactive oxygen species (ROS) clearance.

### 8.4. Other In Vivo Studies with Animal Models

The IL-10 deficient mouse is a classic knockout model that develops spontaneous colitis under pathogen-free conditions. Some authors have explored the influence of GSPE in this model, supplementing colitic animals with 0.1–1 g 100 g^−1^ of dry feed weight for 12–16 days [123,124]. These studies evidenced a reduction of multiple inflammatory markers in the jejunum and colon, such as TNF-α, IL-1β, IL-6, and IFN-γ gene expressions, as well as MPO activity. This anti-inflammatory effect was associated with the inhibition of the NF-κB signaling. Interestingly, GSPE supplementation also increased the density of goblet cells in the jejunum of treated animals, suggesting that there is an alternative mechanism by which inflammation is attenuated.

Cardoso et al. [13] recently tested both dietary (75 mg kg^−1^) and pharmacological doses of GSPE (375 mg kg^−1^) in a rat model of mild intestinal dysfunction induced by intraperitoneal injection of LPS. GSPE was administered daily by oral gavage for 15 days prior to LPS-induced intestinal dysfunction. LPS enhanced intestinal permeability and induced both oxidative stress and inflammation. GSPE-treated animals reduced OVA permeation to the circulation, MPO activity and COX-2 in the small intestine tissues, and reactive oxygen species (ROS) levels in the colon. Furthermore, a gene expression analysis with a low-density microarray technique revealed that unlike the dietary dose of GSPE, the pharmacological dose had a striking effect on the LPS-gene expression profile, showing a strong modulation of multiple genes associated with chemokines and ILs, including upregulation of the anti-inflammatory cytokine IL-13.

### 8.5. Human Ex Vivo Studies

Although the use of animal models is the predominate approximation at preclinical stages for testing novel therapies in intestinal dysfunction, there is a strong trend in the industry towards replacing animal experiments with human cell-culture based models [109,110]. Nevertheless, advantages related to the usefulness of in vitro models for screening of bioactives and exploring action mechanisms are offset by limitations regarding the mimicking of the in vivo situation and translation to the human [133]. Thus, some human ex vivo models have been proposed to test immunomodulatory properties of drug candidates in intestinal explants from IBD patients [134,135]. Intestinal function can also be studied with UCh-based protocols. The UCh system consists of two halves with an opening between them, where mucosal tissue is adapted, thus isolating the apical and basolateral sides of the tissue. This technique has been applied for studying drug absorption [136] and secretion of enterohormones [137] in human endoscopic biopsies. An advantage of UCh models over explant-based models is that UCh models make it possible to measure the electrophysiological parameters, including TEER [136]. All these set-ups permit analyzing the cytokine profiling of intestinal explants or biopsies retaining their in-situ conditioning in a polarized fashion [135,138]. We have employed the UCh to determine TEER and cytokine release (TNF-α) in intestinal tissues from cafeteria diet-induced obese rats treated with GSPE [8,15]. It could also be useful for testing the effect of bioactives on dysfunctional human intestine. A feature of ex vivo models is that screening of drug effects does not compromise the patients by exposing them to unknown outcomes.

### 8.6. Clinical Trials

The use of PACs for intestinal dysfunction is an emerging therapeutic strategy still in preclinical development. There have been no large, well-designed clinical trials that have tested the effectiveness of these phytochemicals. However, current evidence in humans suggests that PAC administration is a promising and safe adjunctive support to current therapies in IBD.

Translation of doses of PAC-rich extracts used in rodent models of intestinal dysfunction to human equivalent doses (HED) indicates that pharmacological doses (up to 5 g d^−1^ for a 60 kg person) could be required to achieve beneficial effects in clinical trials [14,15]. Thus, the first uncertainty involved in assessing the use of PACs as therapy agents in humans is safety. Grape-seed and skin proanthocyanidin-rich extracts have been subjected to toxicological tests in rats to determine their safety for use in functional foods [139,140,141]. In these studies, the median lethal dose (LD50) was found to be greater than 5000 mg kg^−1^ bw (HED > 50 g) when administered once by oral gavage, and 1400–2000 mg kg^−1^ d^−1^ (HED of ≈ 14–20 g d^−1^) was found to be the no-observed-adverse-effect level (NOAEL) for systemic toxicity in sub-chronic administration. A recent study evaluated the safety and tolerability of GSPE intake (up to 2.5 g d^−1^) in a small number of healthy adults for a four-week period and found a good tolerability without adverse effects on hematological or biochemical parameters [47].

To date, there are few clinical studies that evaluate the influence of PACs on intestinal inflammatory conditions. A pilot study was conducted on paediatric Crohn’s disease patients in remission to test the possibility of influencing oxidative stress markers with a procyanidin-rich bark extract from the French maritime pine (Pycnogenol^®^) [142]. Patients showed reduced antioxidant defenses and increased oxidative stress markers at the beginning of the study compared with healthy controls. After 10 weeks of administration (2 mg kg^−1^ d^−1^), Pycnogenol had no impact on inflammatory markers (CRP and calprotectin) and the disease activity index score, but it increased the activity of erythrocyte antioxidant enzymes (SOD and GPx), reduced the level of lipid peroxidation markers in the blood (lipoperoxides and 8-isoprostanes), and reduced protein damage (advanced oxidation protein products). Another clinical study revealed that the postprandial increase of plasma LPS associated with the intake of a high-fat meal was significantly reduced in obese subjects who consumed 1 g of GSPE [143]. As translocation of LPS to the circulation is considered a critical factor in the appearance of systemic low-grade inflammation in patients with metabolic syndrome [144], reduction of postprandial endotoxemia could be particularly interesting from a therapeutic perspective. Large double-blind clinical studies need to be conducted to provide more information on PAC clinical efficacy in intestinal dysfunction so that these phytochemicals can be used therapeutically to improve intestinal health in obese and IBD individuals.

## 9. Biochemical and Molecular Mechanisms Underlying the Barrier Protective and Anti-Inflammatory Properties of PAC in the Intestine

**Figure 3:**
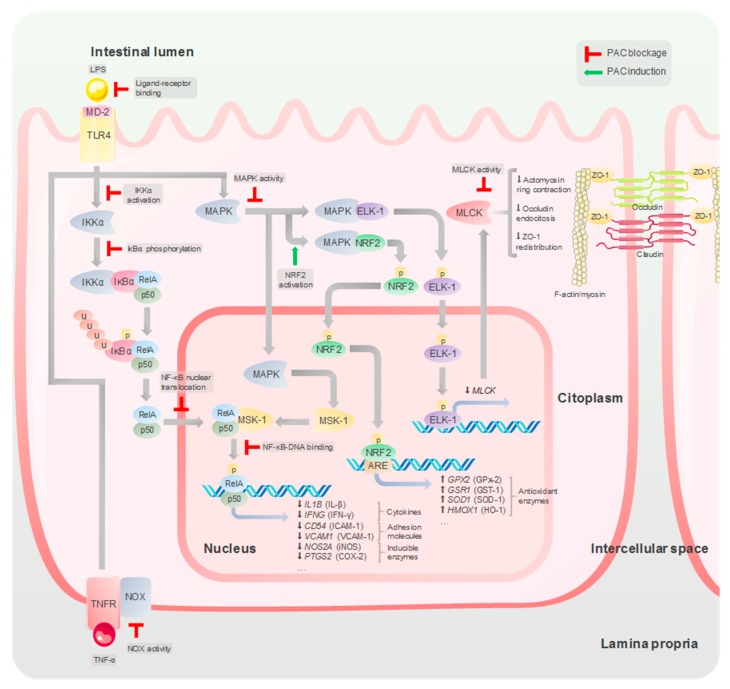
Molecular mechanisms implicated in the physiological effects of PAC in the intestinal mucosa. PACs suppress inflammation interacting with bacterial endotoxins, as well as protein receptors, kinases and transcription factors involved in the pro-inflammatory signaling (NF-κB and mitogen-activated protein kinases (MAPK) pathways). Oxidative stress is mitigated directly by free-radical scavenging and indirectly by the activation of factor-erythroid-2-related factor 2 (NRF2), leading to antioxidant enzyme production via antioxidant responsive element (ARE) binding.

PACs were often considered to be nutritionally undesirable due to their ability to form complexes with macronutrients and reduce the activity of virtually any enzyme implicated in digestion [145,146]. Nevertheless, based on the anti-cancerous, anti-mutagenic, and antimicrobial activities these phytochemicals elicited in laboratory experiments, a role in the modulation of the metabolism and immune system was suggested [146]. The ability of PACs to form cross-links with biomolecules can be attributed to the hydroxyl groups and aromatic rings in their structure that can establish hydrogen bonds and hydrophobic interactions [147]. PACs have a significant affinity for proline-rich proteins and peptides [148]. In general, binding to proteins seems to increase with the DP as larger PAC molecules have more potential binding sites for the associations with proline residues [148]. This phenomenon, responsible for the “tanning effect” that converts animal skin into leather, accounts for most of their therapeutic properties. The interaction results in effects determined by the biological function of the target protein. Thus, PACs not only alter enzymatic activity, but they may also prevent ligand-receptor interactions and the binding of transcription factors to their specific sites in DNA. In addition, some PAC molecules can be adsorbed non-specifically onto biomembrane surfaces [149], affecting their physical characteristics, such as fluidity and density, and potentially altering membrane-dependent processes, including protein receptor activity [150]. Altogether, these effects lead ultimately to the alteration of cell signaling pathways and the modulation of gene expression (Figure 3).

### 9.1. Antioxidant Activity

For a long time, the significance of PACs for promoting overall health was attributed to the antioxidant and free radical scavenging activity shown in many in vitro studies, a hallmark feature of plant phenolic compounds [151]. This property is related to their structure, as the presence of the numerous hydroxyl groups reduces free radicals through electron donation, and the aromatic rings allow the resultant aroxyl radicals to be stabilized by resonance. The antioxidant activity of PACs has been demonstrated in numerous studies performed with PAC-rich foods and derived food products, such as grape, green tea, and cocoa products [152,153]. In particular, GSPEs have shown better free radical scavenging abilities than β-carotene and vitamins C and E [154,155].

New knowledge about the limited bioavailability of PACs meant that the physiological relevance of their antioxidant effects needed to be reconsidered. PACs have been found to have a very limited intestinal absorption [91], and concentrations in the systemic circulation are low with respect to other compounds that display similar antioxidant activity in vivo [133]. Thus, PAC antioxidant activity was unlikely to be relevant for the in vivo situation in several tissues [133], except for those directly exposed to high concentrations, such as the gastrointestinal tract. In vitro studies have suggested that flavan-3-ol and other flavonoids have an inhibitory effect on the NF-κB signaling pathway [156,157]. In addition, it has been demonstrated in HepG2 cells that GSPE improves cell redox status through upregulation of endogenous antioxidant enzymes such as GPx, glutathione S-transferase (GST) and SOD [158]. This is probably mediated by the activation of the nuclear factor-erythroid-2-related factor 2 (NRF2) [159]. These observations support the view of PACs and other polyphenols as versatile bioactives rather than mere antioxidants and encourage the exploration of novel immunomodulatory mechanisms.

### 9.2. Modulation of Signaling Transduction Pathways

PACs exert direct anti-inflammatory effects modulating kinases activity and transcription factors implicated in the production of cytokines, chemokines, and other inflammatory mediators in epithelial and immune cells. Signaling pathways modulated by PACs include NF-κB, ERK1/2 and p38 MAPKs, and NRF2. Park et al. [157] reported an inhibitory effect of monomeric flavan-3-ol and some oligomeric PACs in NF-κB dependent gene expression of IFN-γ-stimulated RAW 264.7 cells (murine macrophages). However, the first demonstration of flavan-3-ols inhibiting NF-κB by direct interaction with key components of the signaling pathway, came from a study by Mackenzie et al. [160]. In this study, Jurkat (human T lymphocyte) cells incubated with (+)-catechin, (–)-epicatechin or B-type dimeric PACs prior to the PMA (NF-κB activator) stimulation showed a reduction in the nuclear translocation of NF-κB due to the inhibition of IKKβ and IκBα phosphorylation. In addition, accumulation of these compounds in the nucleus (especially PAC dimers) dose-dependently suppressed the DNA-binding activity of the p50/RelA complex, indicating that inhibition of the NF-κB signaling pathway also occurred downstream. A subsequent study by the same authors [161] evidenced that dimeric procyanidins B1 and B2, but not A1 or A2, inhibited binding of p50/RelA to DNA. Molecular modelling for these interactions indicated that dimeric B-type PACs adopted a folded structure that mimicked the guanine pair in the κB DNA consensus sequence, which is responsible for the binding of arginine residues in the p50/RelA complex [161]. Terra et al. [11] demonstrated that GSPE also down-regulated IκBα mRNA and inhibited RelA nuclear translocation in RAW 264.7 macrophages stimulated with LPS and IFN-γ. Further in vivo studies confirmed unequivocally that grape-seed PACs orally administered to rats suppressed canonical activation of NF-κB induced by TNBS and a CAF diet in the intestine [120,121] and liver [130].

ERK1/2 and p38 MAPKs and are a family of serine/threonine kinases that mediate cellular responses to external stress signals and cytokines [162]. Activation of p38 MAPK has been associated with transduction of the pro-inflammatory cytokine signal within the intestinal epithelial cell [45]. ERK1/2 and p38 are involved in NF-κB transactivation through the phosphorylation and activation of the mitogen- and stress-activated protein kinase-1 (MSK-1), which in turn phosphorylates RelA [163]. They are also implicated in the phosphorylation of the coactivator p300 required for RelA acetylation [164]. PACs have shown to modulate MAPK activity by different mechanisms. Some authors have suggested that hexameric PACs inhibit bile acid-induced activation of ERK1/2 and p38 MAPKs in intestinal epithelial cells by a lipid raft-dependent effect involving inhibition of NADPH oxidase (NOX) [165]. More recently, a molecular docking analysis indicated that procyanidin B1 may bind to the TLR4/MD-2 complex and be able to act as a competitive antagonist of LPS [166]. This effect was associated with the inhibition of the LPS-induced phosphorylation of p38 MAPK and activation of NF-κB signaling in THP1 (human monocyte) cells. However, PACs may activate ERK1/2 and p38 MAPKs under some oxidative stress situations, which has been associated with translocation of NRF2 to the nucleus. Under oxidative stress, NRF2 promote cell survival by inducing expression of antioxidant enzymes via antioxidant responsive element (ARE) binding [167]. Studies in Caco-2 [159] and HepG2 cells [168] showed that procyanidin B2 and grape-seed PACs induce activation of NRF2, increasing GST [159] and HO-1 [168] activity. More recently, AMPK-induced activation of NRF2 by oral administration of GSPE was associated with a protective effect against lead-induced lung oxidative stress in rats [169].

### 9.3. Modulation of TJ Integrity

The precise mechanisms underlying the improvement in intestine paracellular permeability due to PACs in inflammation are not yet completely elucidated; however, it is known that they lead ultimately to the upregulation (e.g., ZO-1 and claudin-1 [8,13]) or downregulation (e.g., claudin-2 [123]) of TJ protein expression. Loss of TJ integrity in the pro-inflammatory state is mediated by the NF-κB signaling pathway and by the activation of protein kinases MAPKs, PI3Ks, AMPK, and MLCK [43]. MLCK is particularly crucial in actomyosin-based cytoskeletal functions and multiple studies highlight its important role in intestinal TJ remodelling [40,41]. PACs reduce the production of pro-inflammatory mediators (e.g., TNF-α) and reactive oxygen species (i.e., iNOS activity) associated with enhancing intestinal permeability by antagonizing the NF-κB signaling pathway. In addition, PACs are potent inhibitors of kinases including MLCK [43,170]. Contreras et al. [171] suggested that there is an upstream mechanism associated with flavan-3-ols that leads to the prevention of TNF-α-induced intestinal permeability. In this study, TNF-α-stimulated Caco-2 monolayers incubated with (–)-epicatechin showed a reduction of NOX activity, an enzyme that also facilitates activation of TNF-α signaling. This effect was directly associated with the inhibition of ERK1/2 MAPK activity of IκB phosphorylation and of MLCK activation.

### 9.4. Interaction with Bacterial Endotoxins

Delehanty et al. [172] demonstrated that naturally occurring A- and B-type cranberry PACs were able to bind the lipid A moiety of LPS, exhibiting an affinity similar to that of polymyxin B, a potent LPS-binding molecule. In this study, PACs efficiently blocked endocytosis of bacterial LPS in a dose-dependent manner in HEK 293 (human embryonic kidney cells) that expressed receptors TLR4/MD-2 and CD14, thus preventing the induction of the NF-κB signaling pathway without any interaction with cellular components. However, other authors reported that PACs isolated from cocoa beans did not abrogate the binding of LPS to TLR4 in cultivated human dendritic cells [173]. PAC–LPS binding has been linked to the reduction of the post prandial increase in blood LPS associated with the ingestion of a high-fat meal in obese subjects ingesting an oral dose of GSPE [143].

### 9.5. Modulation of Intestinal Microbiota

Dietary PACs, specifically longer polymers, reach the distal intestine nearly intact, where they become fermentable substrates for the commensal microbiota [174]. PACs have been associated with prebiotic properties, boosting the composition of several kinds of probiotics such as *Bifidobacterium* spp., *Lactobacillus* spp. [175] and the stimulator of MUC2 production *Akkermansia muciniphila* [176,177]. Nevertheless, current evidence is somewhat controversial as effects described in different in vivo studies mainly performed with rodents do not always agree. This suggests that interactions between PACs and microbiota depend largely on the botanical source, the types of molecules present in the extracts tested, and the animal model [178].

A recent study by Casanova et al. [179] found that oral administration of GSPE in Wistar rats for eight days resulted in profound changes in the cecal microbiota composition, reducing diversity indices and the ratio of *Firmicutes* to *Bacteroidetes*. Similar results were found in diet-induced obese Sprague Dawley rats supplemented with a PAC-rich extract of the *Pyracantha fortuneana* fruit, although in this study an increase in microbiota diversity was also reported [118]. GSPE supplementation in IL-10 deficient mice resulted in an increased abundance of *Bacteroides* and *Lactobacilli* [123]. Xing et al. [177] reported that the administration of procyanidin B2 in rabbits feeding a high-fat-cholesterol diet, promoted an increase in the relative abundance of *Akkermansia*. These authors proposed that the reduction of metabolic endotoxemia found in animals treated with procyanidin B2 was attributed to the ability of *Akkermansia* to retain the thickness of the intestinal mucus layer, thus reducing intestinal permeability and the leakage of LPS into the circulation [180].

Cuevas et al. [175] found that in vitro fermentation of grape-seed monomers and PACs in human feces resulted in a reduced abundance of *Clostridium histolyticum*. Inhibition of the growth of some infectious microorganisms, such as the mentioned *C. histolyticum* in the intestine and *Helicobacter pylori* in the stomach [181], may be related to the anti-adherence activity that PACs have demonstrated in in vitro studies [182], as adherence to the epithelium is a prerequisite for colonization and infection of the intestinal gastrointestinal mucosa.

Finally, phenolic acids and phenyl-γ-valerolactones resulting from the colonic fermentation of PACs also exhibit a significant bioactivity in cell models and experimental animals [183]. They therefore may partially account for the beneficial anti-inflammatory effects reported in intestinal and peripheral tissues in vivo. Further research is needed to clarify the importance of these microbial products in health promoting properties associated with the intake of PACs.

## 10. Conclusions and Future Perspectives

The health promoting properties of PACs in the intestine are attributed not only to the antioxidant activity inherent to phenolic compounds, but also to the capacity of these phytochemicals to interact with multiple biomolecules, including proteins, biomembrane lipids, and endotoxins. Bioactivity of PACs is highly structure-dependent and enriched botanical extracts composed by a large variety of molecular structures exert a wide range of unrelated physiological effects. In this way, PAC-rich extracts can modulate kinase activity, several signal transduction pathways implicated in the inflammatory response, and the remodelling of TJs. Flavan-3-ol monomers and short PAC oligomers are absorbed by enterocytes and immune cells and exert a direct action on kinases and transcription factors. Bioactivity of larger oligomers and polymeric PACs do not require direct intestinal absorption and are able to bind protein receptors on the enterocyte and immune cell surfaces as well as luminal bacterial endotoxins, thus inhibiting pro-inflammatory signaling and improving barrier integrity. Due to the negligible absorption of large PAC molecules in the short intestine, phenyl-γ-valerolactones and phenolic acids produced by the microbiota metabolism in the colon are thought to play an important role in these health-promoting effects, and thus need to be further researched.

The barrier-protective and anti-inflammatory properties of PACs are emerging as a potential adjunctive support to current therapies for managing obesity related intestinal dysfunction and IBD. However, there have been no large, well-designed clinical trials establishing the efficacy of these phytochemicals in chronic conditions. At preclinical stages, the use of animal models is the predominate approach for testing novel therapies for intestinal dysfunction, although several strategies for replacing animal experiments have been proposed. As there are still no studies on the impact of PACs on human intestinal health, ex vivo models of the human intestine could be a more physiologically reliable alternative to human cell lines and an alternative to animal experimentation in preclinical development.

## Figures and Tables

**Table 1:** Interaction of PACs with permeability and inflammatory markers in cell culture models of intestinal dysfunction.

Extract or Compound	Concentration	Time of Incubation	Cell Line(s)	Permeability and/or Inflammatory Inductor	Outcomes		Ref.
					Permeability/Integrity	Inflammation/Oxidative Stress	
Apple procyanidins	13–50 μg mL^−1^	6 h	Caco-2	PMA (300 ng mL^−1^)	ND	↓ IL-8 release	[112]
Apple procyanidin dimer fraction	50–150 μg mL^−1^	24 h	Caco-2	LPS (50 μg mL^−1^)	↑ Occludin. ↑ ZO-1.	↓ NF-κB and TNF-α gene expression. ↑ GPx, SOD, HO-1.	[10]
Cranberry procyanidins	250 μg mL^−1^	Preincubation for 24 h	Caco-2/15 cells	Fe/Asc mixture (200 μM/2 mM) or LPS (200 μg mL^−1^) for 6 h	ND	↓ PGE_2_ accretion. ↓ COX-2 protein content. ↓ TNF-α and IL-6 protein content.	[113]
Hexameric procyanidins	20 µM	Preincubation for 30 min	Caco-2	TNF-α (10 ng mL^−1^) for 60 min	ND	↓ IκBα phosphorylation. ↓ NF-κB p50 and RelA nuclear translocation. ↓ NF-κB-DNA binding. ↓ iNOS mRNA and protein content ↓ ROS.	[114]
Nut polymeric-PAC fraction	4.8–12 (mg cyanidin equivalents mL^−1^)	Preincubation for 1 h followed by co-incubation for 24 h with the inflammation inductor	Caco-2	IL-1β (25 ng mL^−1^)	↑ TEER. ↓ FSA permeation.	↓ IL-6 and IL-8 release. ↓ IκBα phosphorylation. ↓ RelA nuclear translocation.	[115]
Cocoa procyanidin polymers	100 μg mL^−1^	Preincubation for 24 h	Caco-2	DSS (2% w v^−1^) for 48 h	ND	↓ IL-8 release.	[116]
			HT-29	TNF-α (5 ng mL^−1^) for 6 h	↓ FD (4 kD) permeation	ND	
Procyanidin B2	50 μM	Preincubation for 24 h, co-incubation with the inflammation inductor for a further 48h	Caco-2/HT29-MTX co-culture	LPS-activated Raw264.7 medium	≈ TEER. ↑ Claudin-7. ↑ Occludin. ↓ ZO-1.	ND	[106]
Various PAC-rich extracts (apple and avocado peel, cranberry and grape)	12.5−50 μg mL^−1^	24 h	Caco-2	*p*-Cresol (3.2 mM)	↑ TEER. ↓ FD (4 kD) permeation.	ND	[117]

FSA, fluorescein-5-(and-6)-sulfonic acid trisodium salt. ND, not determined.

**Table 2:** Interaction of PACs with permeability and inflammatory markers in animal models of intestinal dysfunction.

Extract or Compound	Dose (Way of Administration)	Time of Administration	Animal Model	Permeability and/or Inflammatory Inductor	Outcomes		Ref.
					Permeability/Integrity	Inflammation/Oxidative Stress	
GSPE	5, 25 or 50 mg kg^−1^ bw (daily oral gavage)	3 weeks (after 15 weeks of cafeteria diet)	Diet-induced obese Wistar rat	Long-term cafeteria diet (18 weeks)	*Ileum:* ↑ ZO-1 gene expression.	*Ileum:* ↓ IL-1β gene expression. ↓ iNOS gene expression. ↓ MPO activity. ↓ ROS.	[14]
GSPE	500 mg kg^−1^ bw (daily oral gavage)	17 weeks every other week or 10 days (before cafeteria diet).	Diet-induced obese Wistar rat	Long-term cafeteria diet (17 weeks)	↓ Plasma OVA *Duodenum, ileum and colon:* ↑ TEER (ex vivo). *Ileum:* ↑ Claudin-1 gene expression.	*Ileum:* ↓ MPO activity.	[8]
GSPE	100 or 500 mg kg^−1^ bw (daily oral gavage)	2 weeks (after 15 weeks of cafeteria diet)	Diet-induced obese Wistar rat	Long-term cafeteria diet (17 weeks)	↓ Plasma OVA *Ileum and colon:* ↑ TEER (ex vivo). *Ileum:* ↑ Claudin-1 gene expression.	*Duodenum and colon:* ↓ TNF-α release (ex vivo). Ileum: ↓ MPO activity.	[15]
*Pyracantha fortuneana* fruit PAC-rich extract	0.4 or 1 g 100 g^−1^ of dry feed weight (orally)	8 weeks (after second week of high-fat diet).	Diet-induced obese Sprague Dawley rat	High-fat diet (10 weeks)	↓ LMR. ↑ Occludin (segment not specified). ↑ ZO-1 (jejunum).	ND	[118]
GSPE	100, 200 or 400 mg kg^−1^ bw (daily oral gavage)	7 days (after second TNBS-induced colitis)	Wistar rat with TNBS-induced recurrent ulcerative colitis	TNBS (ir. injection of 80 mg kg^−1^, 30 mg kg^−1^ after 16 days)	ND	*Colon:* ↓ TNF-α. ↓ MPO and iNOS activities. ↓ IKKα/β and IκBα phosphorylation. ↓ NF-κB nuclear translocation. ↓ MDA. ↑ GPx and SOD activities.	[119,120]
GSPE	100, 200 or 400 mg kg^−1^ bw (daily oral gavage)	7 days (after TNBS-induced colitis)	Wistar rat with TNBS-induced ulcerative colitis	TNBS (ir. injection of 100 mg kg^−1^)	ND	*Colon:* ↓ IL-1β. ↓ MPO activity. ↓ IKK activity. ↓ IκBα phosphorylation. ↓ RelA protein content.	[121]
Procyanidin B2	10, 20 or 40 mg kg^−1^ (daily oral gavage)	11 days	C57BL/6 mouse with DSS-induced colitis	DSS (2.5 g 100 mL^−1^ of drinking water for 9 days)	ND	*Colon:* ↓ MMP9. ↓ Cleaved caspase-1. ↓ RelA phosphorylation. ↓ TNF-α, IL-1β and IL-6 gene expression.	[122]
GSPE	1 g 100 g^−1^ of dry feed weight (orally)	16 weeks	IL10-deficient mouse prone to colitis	None (spontaneous colitis)	ND	*Colon:* ↓ TNF-α, IL-1β, IL-6 and IFN-γ gene expressions. ↓ MPO protein content and gene expression. ↓ RelA phosphorylation.	[123]
GSPE	0.1 g 100 mL^−1^ of drinking water (orally)	12 weeks	IL10-deficient mouse prone to colitis	None (spontaneous colitis)	ND	*Jejunum:* ↓ TNF-α and IFN-γ. ↑ IκBα protein content. ↑ iNOS gene expression.	[124]
GSPE	75 or 375 mg kg^−1^ bw (daily oral gavage)	15 days (before LPS administration)	Wistar rat with LPS-induced intestinal dysfunction	LPS (ip. injection of 0.3 mg kg^−1^)	↓ Plasma OVA *Duodenum:* ↑ JAM-A gene expression. *Ileum:* ↓ ZO-1, occludin, claudin-2, and JAM-A gene expressions.	*Duodenum:* ↓ COX-2 activity. *Duodenum and ileum:* ↓ MPO activity. *Colon*: ↓ ROS.	[13]

Bw, body weight. LMR, lactulose to mannitol ratio. Ir., intrarectal. Ip., intraperitoneal. ND, not determined.

**Table 3:** Main compounds of the PAC-rich grape-seed extracts (GSPE) used in the in vivo studies on cafeteria (CAF) diet-induced intestinal dysfunction.

Compound	Composition	
	% of Total Flavan-3-ol Content	mg g^−1 ^Extract
Flavan-3-ol monomers	21.3	
Catechin		121.32 ± 3.41
Epicatechin		93.44 ± 4.27
Epicatechin gallate		21.24 ± 1.08
PAC dimers	17.4	
Procyanidin B1		88.80 ± 3.46
Procyanidin B2		33.24 ± 1.39
Procyanidin B3		46.09 ± 2.07
Dimer gallate		8.86 ± 0.14
PAC trimers	16.3	4.90 ± 0.47
PAC tetramers	13.3	0.05 ± 0.01
Other PACs (DP > 5)	31.7	n/a

The GSPE (Vitaflavan^®^) was provided by Les Dérives Résiniques et Terpéniques (Dax, France). According to the manufacturer, the extract has a 75% of procyanidins. HPLC-MS/MS analysis by Margalef et al. Table adapted from [131].
